# Improving Hydrogen Selectivity in Semiconductor Metal Oxide Gas Sensors with Cellulose Nanocrystal Membranes

**DOI:** 10.1021/acssensors.5c01801

**Published:** 2025-10-16

**Authors:** Guglielmo Trentini, Antonio Orlando, Soufiane Krik, Pietro Tosato, Matteo Valt, Luisa Petti, Marina Scarpa, Andrea Gaiardo

**Affiliations:** † Faculty of Engineering, 18956 Free University of Bolzano-Bozen, Via Bruno Buozzi, 1, 39100 Bolzano, Italy; ‡ Materials and Topologies for Sensors and Devices, Sensors and Devices Center, 18466 Bruno Kessler Foundation, Via Sommarive 18, 38123 Trento, Italy; § Department of Physics, University of Trento, Via Sommarive, 14, 38123 Povo-Trento, Italy

## Abstract

As hydrogen (H_2_) gains traction as a clean energy carrier, the need for reliable and selective gas sensors becomes increasingly urgent, particularly for detecting H_2_ leaks amidst complex gas environments. While semiconducting metal oxide (SMOX)-based sensors are attractive due to their low cost and high sensitivity, their poor selectivity remains a major limitation. In this work, we address this challenge by integrating a gas-selective membrane into the sensor packaging, without altering the sensing material itself. The membrane is produced in situ by drop-casting a suspension of cellulose nanocrystals (CNCs) within the sensor housing. The CNCs self-assemble into a thin, gas-permeable barrier upon solvent evaporation. This simple addition significantly reduces cross-sensitivity to common interfering gases, suppressing the response to 30 ppm of ethanol (EtOH), acetone, ammonia (NH_3_), nitrogen dioxide (NO_2_), and carbon monoxide (CO) by factors of approximately 160, 1500, 370, 90, and 20, respectively, while reducing the H_2_ signal by only a factor of 6. The result is a substantial improvement in H_2_ selectivity using a low-cost, scalable approach that preserves the original sensor architecture. This method offers a practical path to enhanced performance in SMOX-based gas sensors for safety and energy applications.

## Graphical abstract

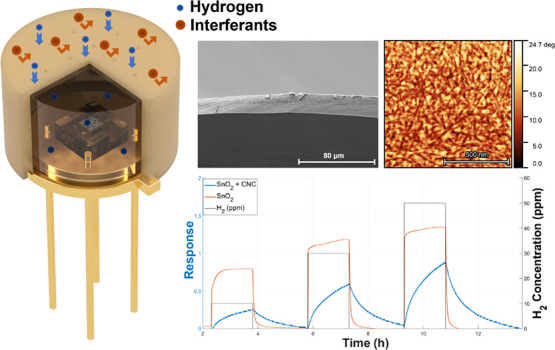

H_2_ is considered a cornerstone of Europe’s decarbonization strategy, serving as a clean energy carrier with the potential to support climate neutrality targets.^1^ However, its integration into existing infrastructure introduces safety challenges due to its high flammability and greater propensity for leakage compared to hydrocarbons.^2^ To minimize these risks and reduce losses during production, storage, transport, and use, continuous monitoring is essential. This underscores the urgent need for low-cost, sensitive, and selective H_2_ sensors capable of enabling widespread deployment and early leak detection.^3^ Semiconducting metal oxide (SMOX)-based gas sensors have long been used in leak detection, offering affordability and high sensitivity. Yet, their poor selectivity remains a critical barrier to reliable real-world application.^4,5^

Most efforts to improve selectivity in H_2_ sensors have centered on modifying the sensing material itself, adjusting the operating temperature, or employing arrays of sensors combined with machine learning algorithms.^6–8^ While each of these strategies can yield performance gains, they also introduce significant challenges that may compromise sensor stability and practicality. For example, functionalizing the active layer with noble metals such as platinum or palladium can enhance sensitivity and selectivity, but often leads to resistance drift over time.^9^ Temperature modulation, another common approach to boosting selectivity, can accelerate sensor degradation and complicate signal processing.^10,11^ Similarly, sensor arrays generate large volumes of high-dimensional, often collinear data, which without careful feature selection can obscure meaningful patterns and lead to overfitting.^12,13^ A common feature across these strategies is that the sensing material remains exposed to both H_2_ and interfering gases, with selectivity achieved by amplifying the relative response of the sensor to H_2_. Although many advanced strategies show promise, their complexity often compromises long-term robustness. In contrast, sensors based on well-established materials like tin dioxide (SnO_2_) offer stable and predictable performance.^14^ Enhancing their selectivity without modifying the sensing layer remains a key challenge, one that can be addressed by introducing a filtering layer upstream of the sensor. Though historically considered a secondary enhancement, selective filtering represents a promising and underexplored route, especially when paired with stable, well-understood sensing platforms.^15,16^ This approach allows for improved performance without compromising the simplicity or reliability of the underlying sensors. Filtering strategies for enhancing gas sensor selectivity typically fall into three main categories: sorption filters, catalytic filters, and size-selective filters, each operating through distinct physical or chemical principles.

Sorption filters improve selectivity by adsorbing or absorbing interfering species, thereby preventing them from reaching the sensing layer. These filters can discriminate analytes based on properties such as polarity, boiling point, hydrophobicity, or molecular weight, and their modular design allows for easy integration into existing sensor systems. However, their performance degrades upon saturation, necessitating periodic regeneration via purging or thermal treatment.^17^ A notable example is the use of activated carbon filters: Berry and Hamwi demonstrated that temperature-controlled activated carbon could selectively remove ozone and NO_2_, thereby enhancing sensor performance in indoor air quality applications.^18^

Catalytic filters function by chemically converting interfering gases into inert or less reactive species before they reach the sensor. This strategy has proven effective in a variety of applications. For instance, Sahm et al. employed flame spray pyrolysis to fabricate multilayer sensors with a Pd/Al_2_O_3_ catalytic overlayer, which selectively oxidized interfering species and improved detection of methane (CH_4_), CO, and EtOH.^19^

Size-selective filters, in contrast, discriminate gases based on their molecular dimensions using microporous membranes or nanostructured films with pore sizes on the order of angstroms. This approach is particularly promising for H_2_ sensing, as H_2_ possesses a small kinetic diameter and high diffusivity, allowing it to pass through restrictive barriers more readily than larger molecules.^20^ Several studies have pursued this strategy by directly coating the sensing material with size-selective layers to block access to larger interferants while allowing H_2_ to permeate.^21,22^ While integrating a membrane directly into the sensor design offers the advantage of a compact form factor, it imposes constraints on the choice of membrane materials. The selected material must be able to withstand the operating temperature of the sensor without degradation.^23^ Furthermore, this integration can limit the range of operating temperatures, as temperature-induced changes in membrane pore size and differences in the coefficients of thermal expansion between the sensing layer and the membrane may lead to device damage or performance degradation.^24^

A promising solution to these challenges is to incorporate the selective membrane into the device packaging, decoupling it thermally from the sensing layer. This approach expands the range of materials that can be used for the membrane, as the material no longer needs to directly withstand the sensor operating conditions of the sensor. For instance, Chen et al. demonstrated the integration of a hydrophobic PTFE membrane into the sensor packaging to block humidity and particulates, thereby enhancing the stability of the sensor in harsh environments and enabling more reliable performance in outdoor and exhaled breath analysis applications.^25^ Similarly, Graunke et al. embedded dense polymer films within the sensor package to act as gas-permeable barriers, facilitating the selective diffusion of small molecules like H_2_, while effectively blocking larger interferents such as acetone and EtOH.^26^

In this study, we employ a gas-selective membrane composed of 2,2,6,6-tetramethyl-1-piperidinyloxy (TEMPO)-oxidized cellulose nanocrystals (CNCs), hereafter referred to as a “CNC membrane” for brevity.^27^ This material was chosen for its unique morphology, excellent gas selectivity, environmental sustainability, low cost, and scalable production process, all of which help maintain a cost-effective device design.^27^ CNCs are needle-like nanoparticles derived from cellulose pulp through a process that separates the crystalline and amorphous regions of the cellulose fibers.^28^ A suspension of CNCs can be easily drop-cast onto the substrate, where it forms a thin film upon solvent evaporation. The high crystallinity, nanoscale dimensions, high aspect ratio, and rigidity of the individual CNCs contribute significantly to the structure and performance of the membrane.^29^ During the drying process, the CNCs self-assemble into a dense, uniform membrane with outstanding gas barrier properties, forming tortuous subnanometer pathways that effectively hinder the diffusion of larger molecules while allowing smaller ones, such as H_2_, to pass through more readily.^30–32^

In this work, a suspension of CNCs is drop-cast into the protective packaging cap of the sensor, where the CNCs self-assemble in situ as the solvent evaporates. Once the membrane is formed, the cap is sealed onto the sensor using an impermeable epoxy resin, ensuring that gas molecules can only reach the sensing layer by diffusing through the membrane. This configuration passively excludes larger interferants that cannot permeate the CNC membrane by molecular diffusion. The integration of the CNC membrane into the sensor platform significantly alters the selectivity profile of the underlying SMOX sensor by modulating gas access to the sensing layer, reducing responses to larger and more polar interferents. This results in a substantial improvement in H_2_ selectivity, with only a minor reduction in the target gas signal. While this enhancement is accompanied by a moderately slower response and recovery time, consistent with the membrane acting as a diffusion barrier, the overall performance remains highly effective. Through systematic analysis of sensor responses and dynamics, the impact of gas molecular properties such as kinetic diameter and dipole moment on permeation behavior has been thoroughly characterized. These results have been validated by analytical permeation studies, confirming the role of the CNC membrane as a passive size- and polarity-selective barrier that favors the transport of smaller, less polar molecules while hindering larger or more polar interferents.

## Materials and Methods

### Synthesis and Fabrication

#### Cellulose Nanocrystal Synthesis by TEMPO-Mediated Oxidation

CNCs were prepared following the protocol of Saito and Isogai from never-dried cellulose pulp (Celeste 85, SCA, Sweden).^33^ Ten grams of pulp were first swelled overnight in 500 mL of distilled (DI) water, then combined with 0.16 g TEMPO and 1.00 g NaBr also dissolved in 500 mL of DI water. Under continuous stirring, 35 mL of NaClO were added to initiate the selective oxidation of the C6 hydroxyl groups to carboxylate groups, maintaining the pH at 10–10.5 for ∼3 h by adding small aliquots of 1 M NaOH. The reaction was stopped once the pH became stable. The slurry was then neutralized and unreacted reagents were removed by six thorough washing steps. The introduction of carboxylate groups aided mechanical fibril separation. The oxidized pulp was sonicated in an ice bath to prevent thermal damage, applying 0.5 W/mL until a total of 1000 W·s/m was reached. The suspension was concentrated at 60 °C until a concentration of ∼5 mg/mL was achieved and finally contaminants and impurities were removed by vacuum filtration over a cellulose acetate membrane with pore size of 5 μm.

#### Sensing Chip Fabrication

The gas sensor device was fabricated using a standard microfabrication process developed at FBK, combined with thick-film deposition of the sensing layer. Detailed fabrication procedures are described elsewhere.^34,35^ Briefly, a 3 × 3 mm^2^ silicon chip (300 μm thick) with a 1.3 × 1.3 mm^2^ suspended MEMS substrate composed of a three layers stack silicon oxide, silicon nitride, and silicon oxide was used as the transduction element.^35^ The triple low-stress supports a 120 nm thick meandering platinum heater and two platinum electrodes. To ensure reproducibility and isolate the effect of the membrane from other mechanisms, commercially available SnO_2_ nanoparticles from Sigma-Aldrich were used. These were chosen for their standardized properties and well-established gas sensing mechanism and were mixed with organic vehicles and ground in an agate mortar to form a homogeneous, screen-printable paste. This paste was then applied onto the suspended MEMS structure to a thickness of approximately 10 μm using an Aurel VS1520A semiautomatic screen-printer. After deposition, the coated devices were calcined at 650 °C for 2 h to remove organic components. Scanning electron microscopy (SEM) and energy-dispersive X-ray spectroscopy (EDX) were performed using a Phenom XL G2 to evaluate the morphology and confirm the removal of organic residues after calcination. SEM imaging revealed a porous network of interconnected nanoparticles, while EDX analysis confirmed the exclusive presence of tin and oxygen in a ratio consistent with SnO_2_ (Figure S4) Finally, the chips were mounted onto standard TO-39 packages and electrically connected via ball-bonding using gold wire.

#### Housing and Membrane Fabrication and Sensor Assembly

The housing was machined from polyether ether ketone (PEEK), chosen for its chemical inertness and low outgassing. One side featured a contour forming a negative imprint of the TO-39 package, ensuring proper alignment and interference fitting. The opposite face was 500 μm thick and contained 12 0.1 μm-diameter pass through holes. To form the CNC membrane, the drilled face was covered with parafilm and placed facing downward. A 300 μL suspension of CNCs was then applied to the interior surface by drop-casting. The assembly was then held under low vacuum for 30 min to remove trapped air and dissolved gases, followed by drying at 40 °C for 8 h, allowing the CNCs to self-assemble into a dense membrane over the perforations. After removing the parafilm, the housing now equipped with the CNC membrane was mounted onto the TO-39 package and sealed with EPO TEK 301 epoxy. Once cured for 24 h, this gastight seal ensured that any gas reaching the sensing element first had to pass through the membrane. The final assembled device, both before and after the membrane cap, is shown in Figure S1.

### Characterization

#### CNC Membrane Characterization

To assess the suitability of CNCs for integration into the gas sensor design, analysis were carried out at different stages of the membrane preparation process. At the suspension stage zeta potential measurements were performed using a Zetasizer Nano ZS. The zeta potential provided insight into the effectiveness of the TEMPO-mediated oxidation by quantifying the surface charge introduced through carboxylic groups, thereby assessing both the degree of functionalization and the colloidal stability of the dispersion. To further investigate the morphology and dimensions of individual nanocrystals, a diluted suspension of CNCs was deposited on a substrate and imaged using atomic force microscopy (AFM). Statistical analysis on 50 measured crystals yielded average values for length and height.

Following membrane casting, further characterization were performed on the solid films. Fourier Transform Infrared Spectroscopy (FTIR), conducted using a Thermofisher Nicolet iN10 Infrared Microscope, was used to verify the presence of carboxylic functional groups introduced during oxidation. Thermogravimetric analysis (TGA) was carried out using a TGA/DSC 2 system from Mettler Toledo under a 50 sccm *N*_2_ flow, employing a 5 °C/min temperature ramp from 30°*C* to 600°*C* to assess thermal stability. Mechanical performance was tested on five membrane samples following ISO 527-3 standards for plastic films, using a Condor Ez Bond Tester (XYZTech) equipped with a 20 N force gauge and a custom clamp to extract average stress and elongation at break. Finally, the morphology of the dried membranes was evaluated by SEM imaging using a Phenom XL G2 Desktop SEM. Surface images were acquired using the secondary electron detector (SED) at 5 kV and 2000× magnification. To observe the internal structure, a membrane was fractured in liquid nitrogen (N_2_) to avoid plastic deformation, allowing clean cross-sectional imaging and visualization of the dense membrane.

#### Sensor Characterization

Two sensors, one with the CNC membrane and one without which served as control, were tested simultaneously. Both originated from the same screen-printing batch and were operated at the same voltage of 3.3 V which corresponds to a working temperature of 350 °C to ensure comparable baseline characteristics.^34^ A more detailed description of the setup can be found in the Supporting Information. The sensor response was quantified using the normalized resistance change, defined as

$$\mathrm{Response}\;(R)=\frac{\mathrm{Resis}\tan{\mathrm{ce}}_{\mathrm{air}}-\mathrm{Resis}\tan{\mathrm{ce}}_{\mathrm{gas}}}{\mathrm{Resis}\tan{\mathrm{ce}}_{\mathrm{gas}}}$$ 1

for reducing gases and

$$\mathrm{Response}\;(R)=\frac{\mathrm{Resis}\tan{\mathrm{ce}}_{\mathrm{gas}}-\mathrm{Resis}\tan{\mathrm{ce}}_{\mathrm{air}}}{\mathrm{Resis}\tan{\mathrm{ce}}_{\mathrm{air}}}$$ 2

for oxidizing gases, where Resistance_gas_ is the resistance measured when the sensor was exposed to the target gas and Resistance_air_ is the resistance in the baseline synthetic air atmosphere. The normalized mean sensor responses and their standard deviations were calculated from the final 2 min of each injection (minutes 28–30), capturing quasi steady-state behavior. All gas measurements were conducted simultaneously in the same controlled chamber, with identical environmental conditions for both the sensor with the CNC membrane and the reference sensor. A constant flow of 200 sccm of dry synthetic air (80% N_2_ and 20% O_2_) was maintained as the baseline atmosphere, and sensors were allowed to equilibrate to a stable baseline resistance before exposure to target gases. Calibration measurements followed a standardized injection protocol. Each analyte was tested at three concentrations: 10, 30, and 50 ppm for EtOH, acetone, H_2_, NH_3_, and CO. For NO_2_, lower concentrations of 1, 3, and 5 ppm were used to account for its strong oxidizing effect, which could saturate the readout electronics at higher levels. These levels were chosen either to remain below the 8 h OSHA permissible exposure limit (TLV-TWA), or because they correspond to concentrations typically encountered in our target applications, ensuring the relevance of the tests to real-world conditions.^36^

This protocol, summarized in Table 1, ensured consistent testing across analytes while accommodating both sensor limitations and practical considerations.

**Table 1:** OSHA 8 h Permissible Exposure Limits (TLW-TVA) and Tested Concentration Ranges for Analyte Gases^36^

gas	OSHA TLV-TWA (ppm)^a^	tested range (ppm)^b^
EtOH C_2_H_5_OH	1000	10, 30, 50
acetone ((CH_3_)_2_CO)	250	10, 30, 50
NH_3_	25	10, 30, 50
CO	50	10, 30, 50
H_2_	*no OSHA limit*	10, 30, 50
NO_2_	5	1, 3, 5

a OSHA permissible exposure limits (TLV-TWA) for an 8 h time-weighted average.

b Tested ranges during calibration measurements.

Each injection lasted 30 min, followed by a 120 min recovery in synthetic air to restore the baseline. This consistent protocol ensured reliable comparison between the sensor with the CNC membrane and the reference sensor in order to more accurately illustrate the impact of the membrane on selectivity.

#### Gas Permeability Analysis

Gas permeability through the CNC membranes was characterized using a custom constant pressure–variable volume setup based on the design described by Fraga et al.^37^ The system incorporates a permeation cell in which the CNC membrane serves as the barrier between two independently controlled gas streams. The configuration is illustrated in Figure S2.

On the retentate side, a constant flow of 100 sccm N_2_ was maintained. On the permeate side, a significantly lower flow of 1 sccm N_2_ was used to carry permeated gases to a quadrupole mass spectrometer (QMS) for detection. This reduced flow was chosen to increase analyte concentrations at the detector and compensate for the limited sensitivity of the QMS.

To begin each measurement, both sides of the setup were flushed with the carrier gas to establish a clean baseline. Then, the retentate side was switched from carrier to the analyte gas mixture using a pressure controller, maintaining a 3 bar pressure difference across the membrane. The gases tested were helium (He), N_2_, carbon dioxide (CO_2_), oxygen (O_2_), and H_2_, with respective concentrations of 100, 100, 40, 20, and 4% (N_2_ being the complementary gas where applicable). For N_2_ measurements, He was used as the carrier gas to avoid detection overlap.

Permeability was determined by imposing a step change in analyte concentration on the retentate side and monitoring the resulting steady-state signal on the permeate side via the QMS. While the setup could in principle be used to extract diffusivity, this was not possible under the present conditions. The relatively thick CNC membranes (∼5 μm) were required to sustain the pressure gradient, which significantly reduced permeation rates. Consequently, the slow rise in signal, combined with the need to reduce carrier flow to 1 sccm to ensure detectability, introduced a time lag comparable to the dead time of the QMS. As a result, only steady-state permeability values could be reliably extracted from these experiments.

## Results and Discussion

### CNC Membrane Characterization

To assess the suitability of the CNC membrane for integration with the sensor, the CNCs were first evaluated in suspension. The full analysis performed are discussed in the Supporting Information. The dispersion exhibited excellent colloidal stability, with a strongly negative zeta potential of −64.7 ± 3.5 mV, consistent with the successful introduction of negatively charged carboxylic groups through TEMPO-mediated oxidation. Mechanical testing of the cast membranes revealed a stress at break of 141 ± 17 MPa and an elongation at break of 1.3 ± 0.5%, confirming that the membrane possesses sufficient mechanical strength for handling and withstanding pressure differentials typical of sensor operation (Figure S3b). Thermal stability was verified via TGA, which showed a mass loss of 10.3% due to dehydration and a degradation onset at 236.55 °C, well above the temperatures encountered within the sensor assembly (Figure 1a).

**Figure 1:**
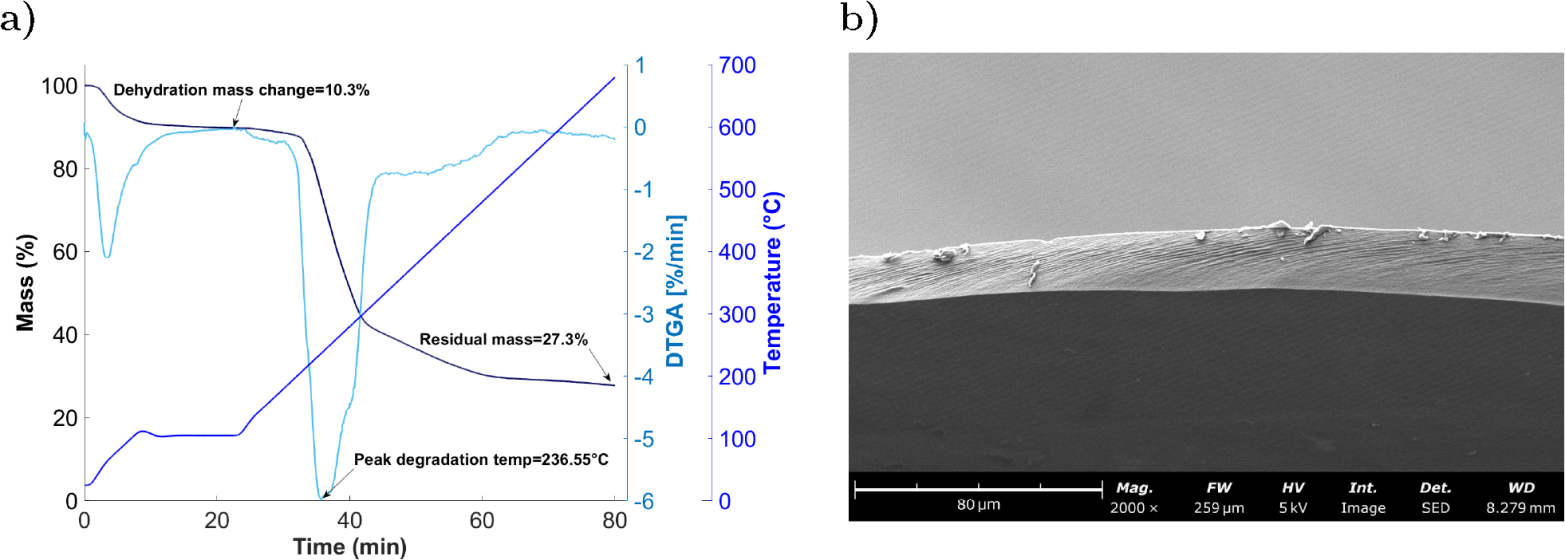
(a) Thermogravimetric analysis (TGA) of the CNC membrane, highlighting the mass change of 10.3% due to dehydration and the peak degradation temperature of 236.55 °C. (b) Cross-sectional view of the CNC membrane obtained by SEM after fracture in liquid N_2_, showing a dense and uniform internal structure.

The morphology of the cast CNC membrane was further assessed by SEM. Surface imaging revealed a flat, featureless topology indicative of uniform self-assembly, while a cross-sectional view obtained after fracturing the membrane in liquid He confirmed a compact and homogeneous internal structure with no visible porosity at these length scales (Figure 1b). These results attest to the dense packing of CNCs during drying, forming CNC a continuous and uniform membrane.

To complement these morphological and thermal characterizations, Figure 2a presents the FTIR spectrum of the CNC membrane, showing a characteristic carbonyl stretching peak at 1610 cm^–1^ that confirms the introduction of carboxylate groups via TEMPO oxidation.^38^ Together with the negative zeta potential measured in suspension, this supports the presence of surface charge, which stabilizes the colloid and, in combination with residual hydroxyl groups, contributes to the overall polarity of the membrane and to the extensive network of hydrogen bonds among the CNCs.

**Figure 2:**
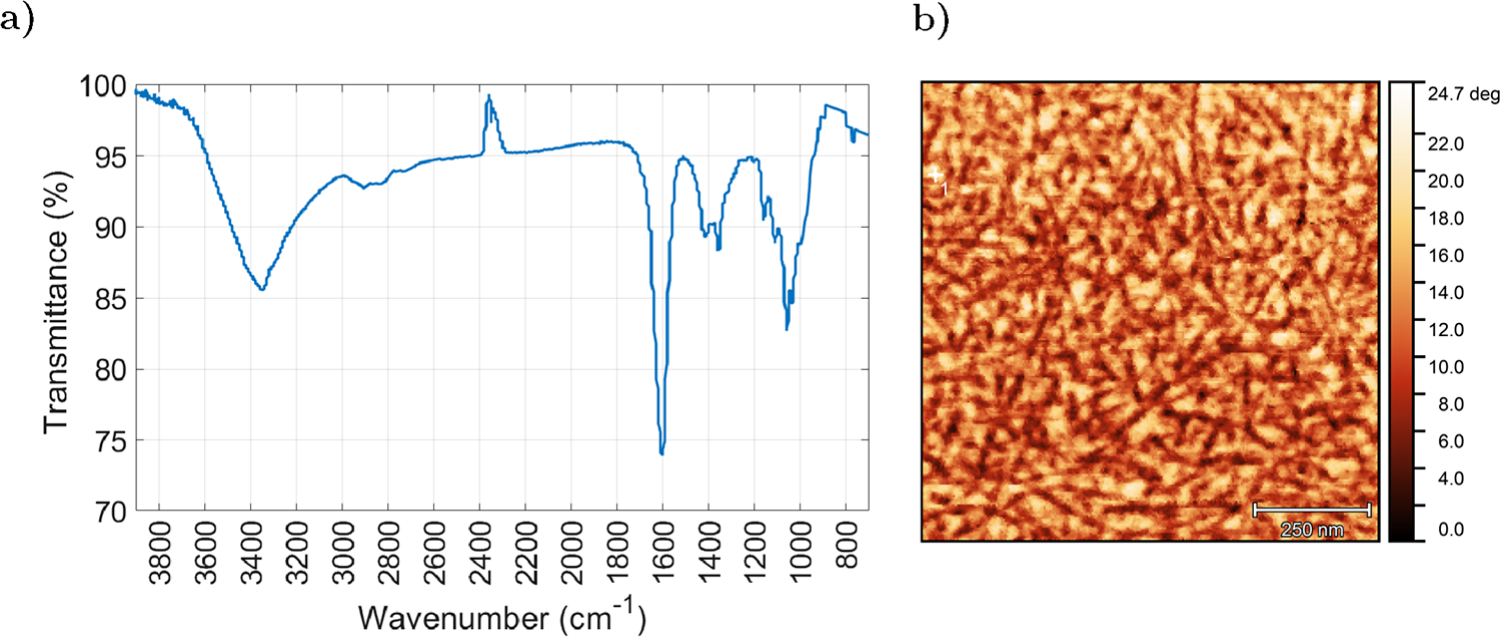
(a) FTIR spectrum of the CNC membrane, showing the characteristic peak around 1610 cm^–1^, confirming the successful introduction of carboxyl groups via TEMPO-mediated oxidation. (b) AFM image (phase mode) of the CNC membrane surface, highlighting the disordered, isotropic arrangement of nanocrystals. While phase imaging does not provide height information, the needle-like CNCs and interstitial voids are clearly visible.

In addition to the cross-sectional SEM presented in Figure 1b, the membrane surface was also examined. However, SEM imaging revealed a completely smooth and featureless morphology. To obtain higher-resolution structural information, AFM was employed over a 1 μm × 1 μm area. The image shown in Figure 2b was acquired in phase mode to enhance lateral contrast, revealing a disordered, densely packed arrangement of CNCs with clearly visible voids between them. This confirms that at the nanoscale level the membrane is not a continuous polymeric film, but rather a stacked network of individual nanocrystals.

To extract morphological parameters for the individual CNCs themselves, we analyzed dilute samples drop-cast onto flat substrates. In this configuration, isolated nanocrystals could be identified against the background and measured directly. From this analysis, we obtained an average length of 206 ± 50 nm and a height of 5.6 ± 1.4 nm, consistent with individualized rod-like particles.

Overall, these characterizations confirm that CNCs in suspension have high colloidal stability and self-assemble forming membranes having dense morphology at the microscale, high mechanical integrity and thermal robustness. AFM imaging further reveals that, as expected, the membrane consists of a randomly packed network of needle-like nanocrystals at the nanoscale. Taken together, these features make the membrane suitable for integration into into SMOX-based gas sensors.

### Gas Sensor Data

Figure 3 shows a typical dynamic response of a pristine SnO_2_ sensor, hereafter referred to as the SnO_2_ sensor or the pristine sensor, during repeated acetone exposures, selected as a representative case to illustrate the behavior of SMOX sensors toward VOCs.

**Figure 3:**
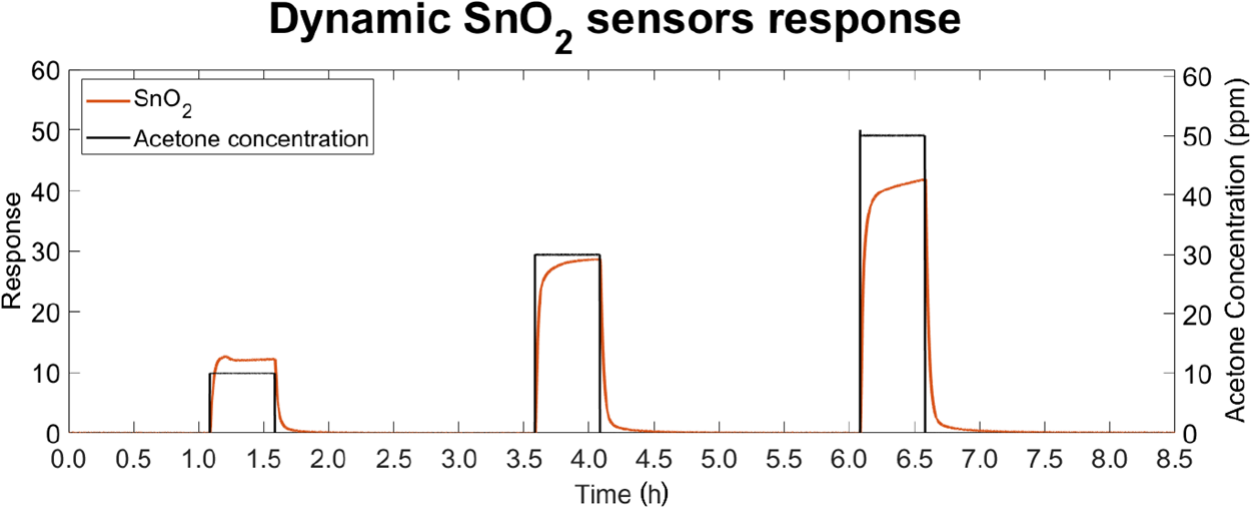
Dynamic response of a sensor employing SnO_2_ as the gas-sensitive layer during acetone injection. The secondary *y*-axis shows acetone concentration, with each injection lasting 30 min, followed by a 120 min recovery period.

The dynamic response observed upon exposure to VOCs is consistent with typical SMOX sensor behavior. To ensure the response profile remains consistent with expected SMOX behavior across different analytes, calibration curves were obtained and are shown in Figure 4. The sensor shows the expected trend: high sensitivity to polar volatile organic compounds such as EtOH and acetone, moderate responses to reducing gases like NO_2_ and H_2_, and a lower but measurable sensitivity to NH_3_ and CO.^35^ These results serve as a baseline for assessing the impact of the CNC membrane on sensor selectivity.

**Figure 4:**
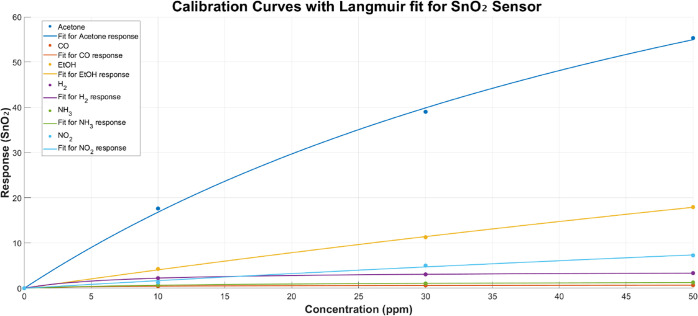
Calibration curves of the pristine SnO_2_ sensor for all tested gases, showing normalized sensor response as a function of concentration. The data confirm the expected gas sensitivity profile characteristic of SnO_2_ sensors.

Since the CNC membrane reduces the response to all gases except H_2_, a direct comparison between the responses of the reference sensor and the membrane-integrated sensor is visually challenging, thus the mean response and standard deviation data is provided in Table 2. The data clearly show a substantial reduction in response for most interfering gases when the membrane is applied, while the response to H_2_ remains comparatively high.

**Table 2:** Normalized Responses and Standard Deviations for All Tested Gases and Concentrations, Comparing the Sensor with the CNC Membrane (SnO_2_ + CNC) to the Reference Sensor without the Membrane (SnO_2_)

gas	concentration (ppm)	response SnO_2_ + CNC	response SnO_2_
acetone	10	0.017 ± 0.002	17.6 ± 0.01
	30	0.026 ± 0.002	39 ± 0.01
	50	0.032 ± 0.002	55.3 ± 0.05
CO	10	0.021 ± 0.002	0.458 ± 0.006
	30	0.028 ± 0.002	0.62 ± 0.005
	50	0.03 ± 0.002	0.689 ± 0.006
EtOH	10	0.03 ± 0.002	4.25 ± 0.03
	30	0.07 ± 0.002	11.3 ± 0.05
	50	0.095 ± 0.002	17.9 ± 0.06
H_2_	10	0.254 ± 0.002	2.24 ± 0.005
	30	0.55 ± 0.01	3.08 ± 0.005
	50	0.76 ± 0.06	3.36 ± 0.004
NH_3_	10	0.0013 ± 0.0001	0.678 ± 0.004
	30	0.0029 ± 0.0001	1.06 ± 0.007
	50	0.0093 ± 0.0001	1.27 ± 0.008
NO_2_	1	0.0194 ± 0.004	1.29 ± 0.01
	3	0.053 ± 0.006	5 ± 0.2
	5	0.072 ± 0.005	7.3 ± 0.2

To better visualize the response of the sensors and their sensitivity to specific analytes, the sensor average response for each concentration were plotted as radar graphs. Each gas is represented on one of the six vertices, and the corresponding normalized sensor response at that concentration is shown as the radial value. This provides an intuitive way to compare sensor performance across different gases and concentrations.

From the radar plots (Figure 5), the reference SnO_2_ sensor shows particularly high responses to EtOH and acetone, while offering more moderate sensitivity to CO and NO_2_, and a comparatively low response to H_2_. By contrast, integrating the CNC membrane drastically suppresses signals from these interfering gases. For example, at 50 ppm the response to acetone drops from 55 in the pristine sensor to 0.03, and that of EtOH from 18 to 0.095. Despite this broad suppression, the SnO_2_ + CNC sensor still exhibits a relatively strong response to H_2_ (0.76 at 50 ppm), indicating that the membrane selectively impedes certain gases while allowing others to permeate and interact with the SMOX sensor. Table 3 provides the kinetic diameters and dipole moments of the tested gases to help shed light onto change of response pattern of the sensor with the CNC membrane compared to the reference.

**Figure 5:**
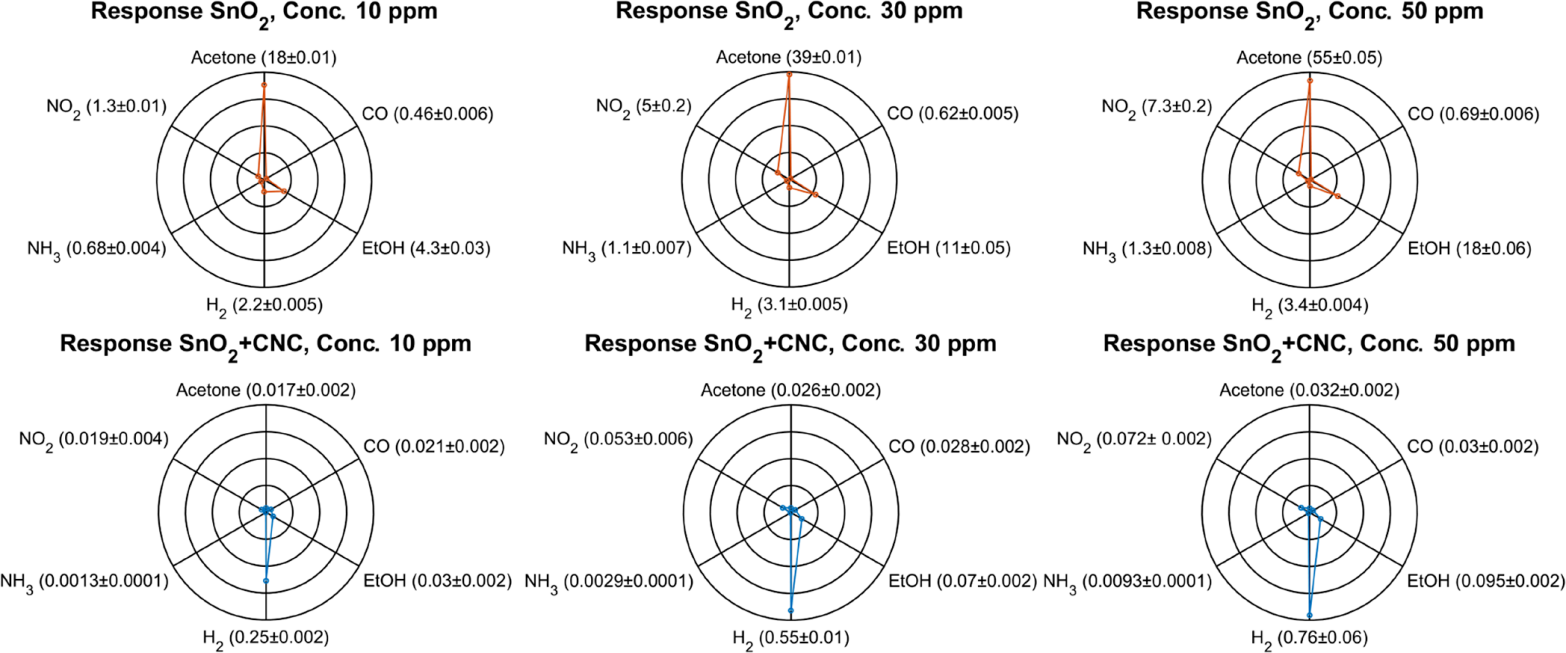
Radar plots compare SnO_2_ and SnO_2_ + CNC sensor responses to six gases (acetone, CO, EtOH, H_2_, NH_3_, and NO_2_) at three concentrations (10, 30, and 50 ppm). For NO_2_, lower concentrations (1, 3, and 5 ppm) were used to avoid saturating the readout electronics due to its strong oxidizing effect on resistance. The SnO_2_ sensor shows high sensitivity to acetone and EtOH, while the SnO_2_ + CNC sensor responds mainly to H_2_.

**Table 3:** Kinetic Diameters and Dipole Moments of the Tested Gases, Ordered By Size^39^

gas	kinetic diameter (Å)	dipole moment (*D*)
NH_3_	2.60	1.47
H_2_	2.89	0.00
NO_2_	3.30	0.63
CO	3.76	0.112
EtOH (C_2_H_5_OH)	4.40	1.36
acetone (C_3_H_6_O)	4.60	2.91

As shown in Table 3, NH_3_ has the smallest kinetic diameter (2.60 Å), smaller than H_2_ (2.89 Å), which permeates through the CNC membrane and elicits strong responses. Based on size alone, NH_3_ would be expected to permeate through the membrane even more readily. However, the SnO_2_ + CNC sensor exhibits a significantly reduced response to NH_3_, suggesting that chemical interactions within the membrane play a more prominent role than size exclusion. To illustrate this, Figure 6 shows the dynamic sensor signal of both the SnO_2_ and SnO_2_ + CNC sensors to NH_3_ at concentrations of 10, 30, and 50 ppm.

**Figure 6:**
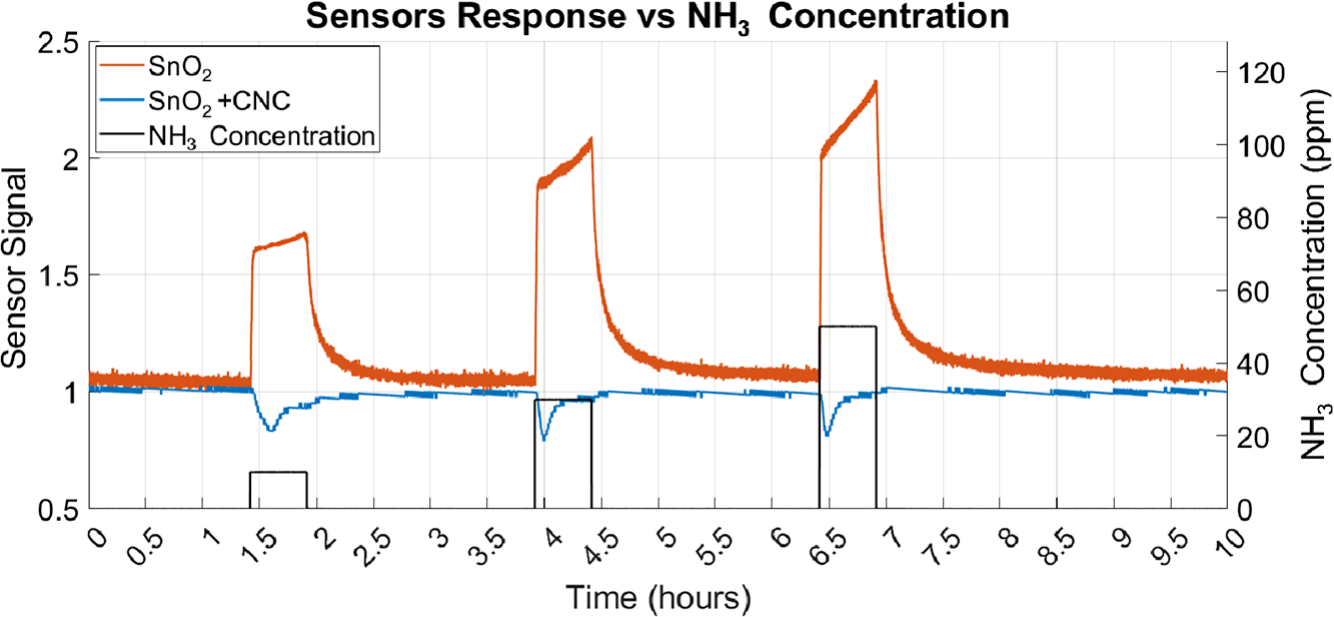
Dynamic sensor signal of SnO_2_ and SnO_2_ + CNC sensors to an injection NH_3_ at 10, 30, and 50 ppm. Note the transient oxidizing behavior in the SnO_2_ + CNC sensor during the initial phase of each injection.

As negative responses have no physical meaning the behavior of the two sensors shown in Figure 6 is calculated from the measured resistance as

$$\mathrm{Sensor}\;\mathrm{Signal}=\frac{\mathrm{Resis}\tan{\mathrm{ce}}_{\mathrm{air}}}{\mathrm{Resis}\tan{\mathrm{ce}}_{\mathrm{gas}}}$$ 3

Thermogravimetric analysis provides insight into this effect, revealing that the CNC membrane retains approximately 10 wt % of water under ambient conditions (Figure 1b). Additionally, TEMPO-oxidized CNCs are known to form hydrogen bonds and electrostatic interactions with ammonium species, which may further influence NH_3_ transport and retention within the membrane.^40^

A plausible mechanism involves a competitive substitution process at the membrane surface, whereby adsorbed water molecules are displaced by incoming NH_3_.^41^ This interaction may transiently block or saturate permeation pathways, reducing the amount of NH_3_ reaching the sensing layer, and at the same time leading to the desorption of H_2_O molecules from the membrane. Once the NH_3_ is flushed out and synthetic air is reintroduced, water gradually redistributes within the membrane, restoring its initial properties. The consistent reappearance of the transient oxidizing signal in subsequent injections supports the hypothesis of a reversible, water-mediated interaction that temporarily modulates gas transport.

Since cross-sensitivity provides a direct comparison between target and interfering responses, it serves as a useful indicator for understanding how the membrane modifies sensor performance. For this purpose, the cross-sensitivity at different concentrations was calculated for both the SnO_2_ and the SnO_2_ + CNC sensors as

$$\mathrm{Cross}-\mathrm{sensitivity}\;(C)=\frac{{\mathrm{Response}}_{\mathrm{gas}}}{{\mathrm{Response}}_{H_{2}}}$$ 4

From the radar plots (Figure 7), it is evident that the SnO_2_ sensor exhibits high cross-sensitivity, showing significant response to several interfering gases. Its relatively high response to VOCs, such as acetone and EtOH, increases more rapidly than the response to H_2_, especially at higher concentrations.

**Figure 7:**
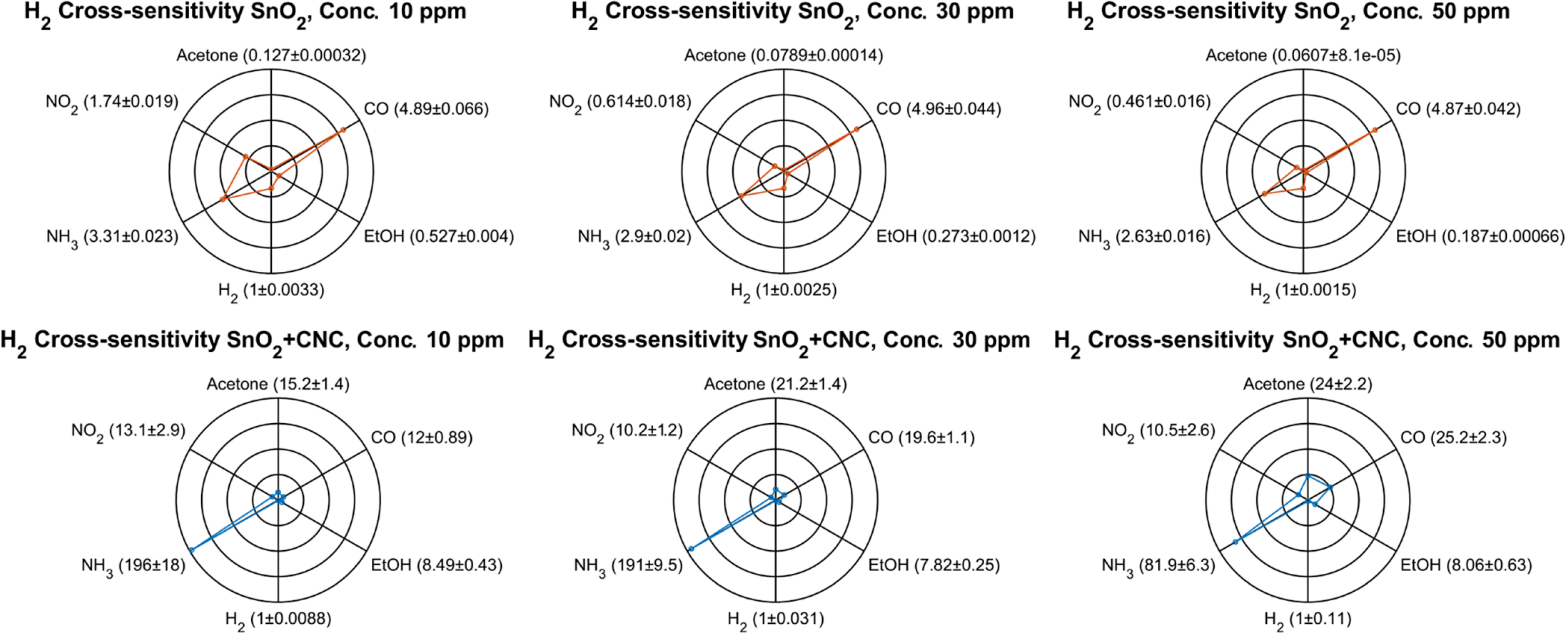
Polar plots showing the cross-sensitivity of H_2_ relative to other gases at the tested concentrations. For NO_2_, the cross-sensitivity was calculated with 1, 3, and 5 ppm.

By contrast, the SnO_2_ + CNC sensor shows a general reduction in the absolute response to interfering gases while retaining a stronger response to H_2_, resulting in improved selectivity. For instance, the response to acetone grows more slowly with concentration than H_2_, suggesting lower acetone permeability through the CNC membrane. EtOH shows a similar cross-sensitivity trend across concentrations, indicating an increase in response comparable with H_2_.

CO yields a nearly parallel response trend to H_2_ in the pristine sensor, resulting in steady cross-sensitivity. In the SnO_2_ + CNC device, however, the CO response increases more slowly than H_2_ as concentration rises, increasing in selectivity at higher concentrations.

NH_3_ elicits a moderate response in the SnO_2_, which increases more with concentration compared to H_2_. The SnO_2_ + CNC sensor initially suppresses the NH_3_ response at low concentrations, but this suppression weakens at higher doses—possibly due to diminishing transient oxidizing behavior observed during exposure.

For NO_2_, the SnO_2_ shows a rapidly increasing response with concentration, outpacing the H_2_ signal and reducing selectivity. In contrast, the SnO_2_ + CNC sensor maintains a more constant response to NO_2_ and H_2_ across concentrations.

Although cross-sensitivity as a metric offers useful insight into gas permeation, it cannot decouple the effects of the membrane from the intrinsic selectivity of the SnO_2_ sensor. It combines both the selective permeability of the membrane and the intrinsic selectivity of the sensor, making it insufficient to isolate the membrane induced H_2_ selectivity enhancement.

For this reason, we measured the barrier effect (*B*), defined as

$$\mathrm{Barrier}\;\mathrm{effect}\;(B)=\frac{{\mathrm{Response}}_{{\mathrm{SnO}}_{2}}^{\mathrm{Gas},\mathrm{Concentration}}}{{\mathrm{Response}}_{{\mathrm{SnO}}_{2}+\mathrm{CNC}}^{\mathrm{Gas},\mathrm{Concentration}}}$$ 5

Here, Response_SnO_2__^Gas,Concentration^ represents the response of the SnO_2_ sensor for a given gas at a specified concentration, and Response_SnO_2_+CNC_^Gas,Concentration^ represents the response of the sensor with the CNC membrane in the same conditions. The parameter *B* quantifies the decrease in the response to each interfering gas produced by the presence of the CNC membrane. Indeed, larger *B* values indicate a more effective suppression of the sensor response to a specific gas.

The barrier effect plots (Figure 8) demonstrate clear trends in how the CNC membrane suppresses different gases based on their physicochemical properties. Acetone shows the strongest suppression, with the barrier effect increasing from over 1000 at 10 ppm to nearly 1700 at 50 ppm. This is consistent with its large kinetic diameter and high dipole moment, which likely hinder diffusion through the membrane and promote stronger interactions with polar groups on the CNCs surface.

**Figure 8:**
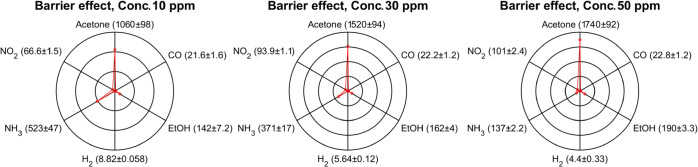
Polar plots showing the barrier effect (*B*) for different gases at the three tested concentrations, highlighting the high barrier effect toward VOCs and NH_3_. For NO_2_, the (*B*) was calculated with 1, 3, and 5 ppm.

EtOH follows a similar trend, with increasing suppression at higher concentrations. NH_3_ also shows strong attenuation, especially at low concentrations (barrier effect >500 at 10 ppm), but this decreases as concentration increases. This decline aligns with the reduced influence of the transient oxidative effect discussed earlier, where initially NH_3_ dissolution in membrane-retained water leads to oxidative behavior that further suppresses its signal, but this effect weakens with repeated or higher-dose exposures.

NO_2_, although smaller than VOCs, is still significantly suppressed by the membrane. Its barrier effect increases modestly with concentration, suggesting that its higher dipole moment plays a more important role than size in governing its interaction with the membrane. CO, with lower polarity and moderate size, shows weaker and relatively constant suppression. As expected, H_2_ exhibits the lowest barrier effect across all concentrations, with a slight decreasing trend, confirming its minimal interaction with and easy permeation through the CNC membrane. A more detailed discussion of the barrier effect trends across concentrations and of the limit of detection is presented in Figures S5 and S6.

Figure 9 displays the barrier effect at 50 ppm for the tested gases, with kinetic diameter on the *x*-axis and dipole moment on the *y*-axis. The heatmap uses a color scale to represent the magnitude of suppression by the CNC membrane, where warmer colors indicate a stronger barrier effect.

**Figure 9:**
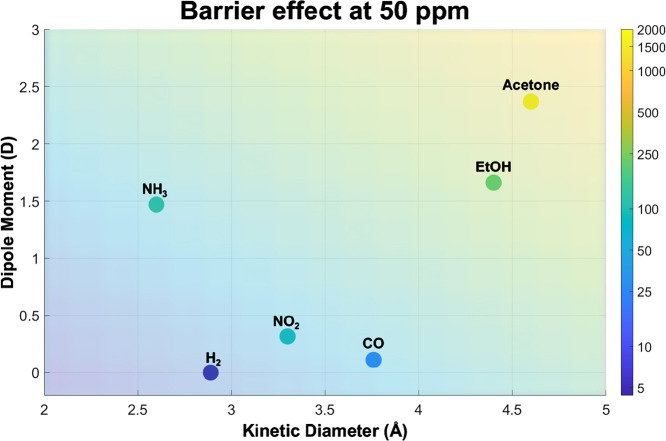
Barrier effect at 50 ppm as a function of kinetic diameter and dipole moment, visualized as a 2D heatmap showing an increase in barrier effect with dipole moment and kinetic diameter.

Gases with larger kinetic diameters and higher dipole moments, such as acetone, are found at the upper end of the color range and in the top right of the graph, corresponding to the most pronounced suppression. In contrast, smaller and weakly polar gases like H_2_ and CO lie at the bottom left of the graph and exhibit exhibiting minimal suppression. This 2D heatmap clearly suggests that molecular size and polarity play key roles in determining the filtering behavior of the membrane, offering a predictive tool for assessing the attenuation of other analytes at 50 ppm.

The addition of the CNC membrane alters sensor dynamics by introducing a transport barrier that affects how quickly gases reach and leave the sensing layer. These effects are quantified using the response (*T*_90_) and recovery times (*T*_10_), which correspond to the time required to reach 90% of the steady-state response during gas exposure and 10% of the baseline during recovery, respectively. Full numerical values are available in Figure S7; here the focus will be on relative trends across gases and concentrations.

Figure 10 shows the relative increases in *T*_90_ and *T*_10_ caused by the CNC membrane. In general, both response and recovery times increase across all gases, with the extent of the delay strongly dependent on gas properties and concentration. For *T*_90_, H_2_ shows the most pronounced increase with concentration, suggesting a diffusion-limited process where the membrane increasingly delays the response at higher doses. CO and EtOH follow similar trends, though with more moderate increases. Acetone, by contrast, shows a relatively constant *T*_90_ across concentrations, indicating a strong limiting interaction already present at low concentrations that does not intensify further with dose. NO_2_ behaves differently, showing a high *T*_90_ at 1 ppm that decreases at higher concentrations. This may be due to its strong dipole moment, which leads to strong interactions with the membrane. These interactions likely cause a rapid occupation of available adsorption or reactive sites, after which diffusion through the membrane becomes less hindered

**Figure 10:**
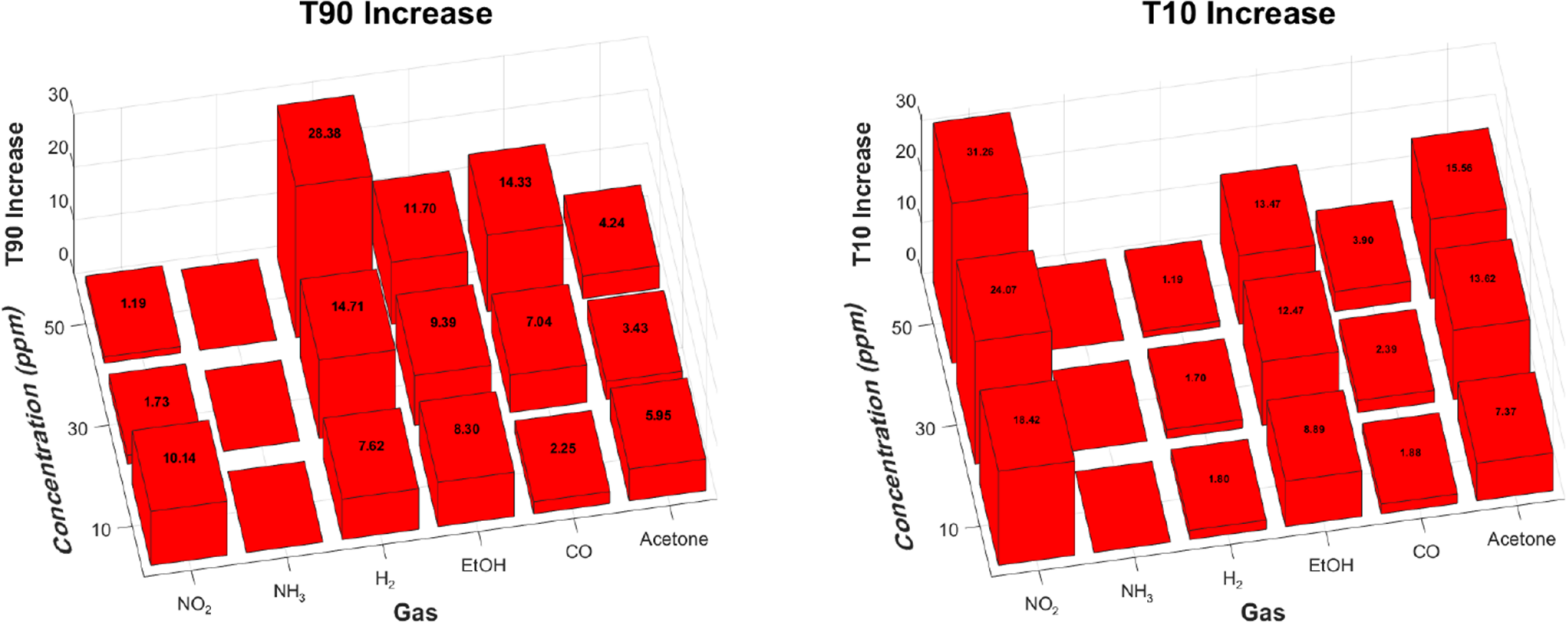
Relative increases in *T*_90_ (left) and *T*_10_ (right) for the SnO_2_ + CNC sensor compared to the SnO_2_ sensor across different gases and concentrations. NO_2_ actual concentrations are 1, 3, and 5 ppm, here, are shown together with all other gases for visual clarity.

*T*_10_ also increases with concentration for most gases, driven by slower desorption and restricted diffusion. The largest rise is seen for NO_2_, likely due to its strong dipole interactions, which play a relatively larger role at low concentrations during desorption. Acetone and EtOH show similarly high *T*_10_ increases, consistent with their polarity and size. CO and especially H_2_ exhibit smaller increases, reflecting weaker interactions and faster exit from the membrane.

Overall, these trends show that while the CNC membrane enhances selectivity, it also slows sensor dynamics. The extent of this delay depends on molecular size and polarity, with more interactive analytes exhibiting longer retention during both response and recovery.

This trade-off might be not only acceptable, but beneficial for leak detection scenarios, where reliable identification of hydrogen is more important than absolute response speed. The enhanced selectivity reduces cross-sensitivity and false positives, allowing lower and more confident thresholds to be set for triggering alarms. This in turn enables earlier detection of genuine leaks, even if the sensor’s time to full equilibrium is slightly longer. In real-world deployment, physical factors such as sensor placement can have a much greater impact on detection latency than a few minutes of response delay. For example, installing sensors near the ceiling takes advantage of hydrogen’s low density to catch leaks rapidly. Additionally, time-to-alarm can be further reduced by analyzing the rate of signal change rather than waiting for steady-state values, as demonstrated in prior work on dynamic response strategies.^42^

The addition of the CNC membrane reshapes the selectivity profile of the SnO_2_ sensor, strongly suppressing the response to larger, polar molecules while preserving much of the signal from smaller or less interactive gases. VOCs like acetone and EtOH are the most attenuated, whereas H_2_ maintain substantial responses. This shift is consistent with the filtering mechanism of the membrane, which favors the permeation of smaller, less polar species. As a result, the membrane-modified sensor reduces cross-sensitivity to interfering VOCs, enabling more reliable discrimination between target and background gases despite slower overall dynamics.

### Cellulose Nanocrystals Permeation Properties

To gain insight into the permeability of the CNC membrane under realistic operating conditions, the response of the SnO_2_ sensor can be used to infer the gas concentration inside the housing of the SnO_2_+CNC device. By analyzing both the response magnitude and the *T*_90_ time, it is possible to estimate the flux of gas permeating through the membrane. This section focuses on H_2_, as it is both the primary target analyte and the only gas observed to permeate the membrane effectively. Combined with the known geometry of the membrane housing, this approach allows the extraction of an effective permeability coefficient, which is later compared with values obtained from direct analytical measurements. An H_2_ calibration curve was established for the SnO_2_ sensor by measuring its steady-state response as a function of known H_2_ concentrations, as detailed in the previous section. The resulting data were fitted with a Langmuir-type isotherm, yielding a functional relationship between response and analyte concentration. This allowed the H_2_ concentration inside the membrane housing of the SnO_2_+CNC sensor to be calculated from its observed response, as shown in Figure 11a.

**Figure 11:**
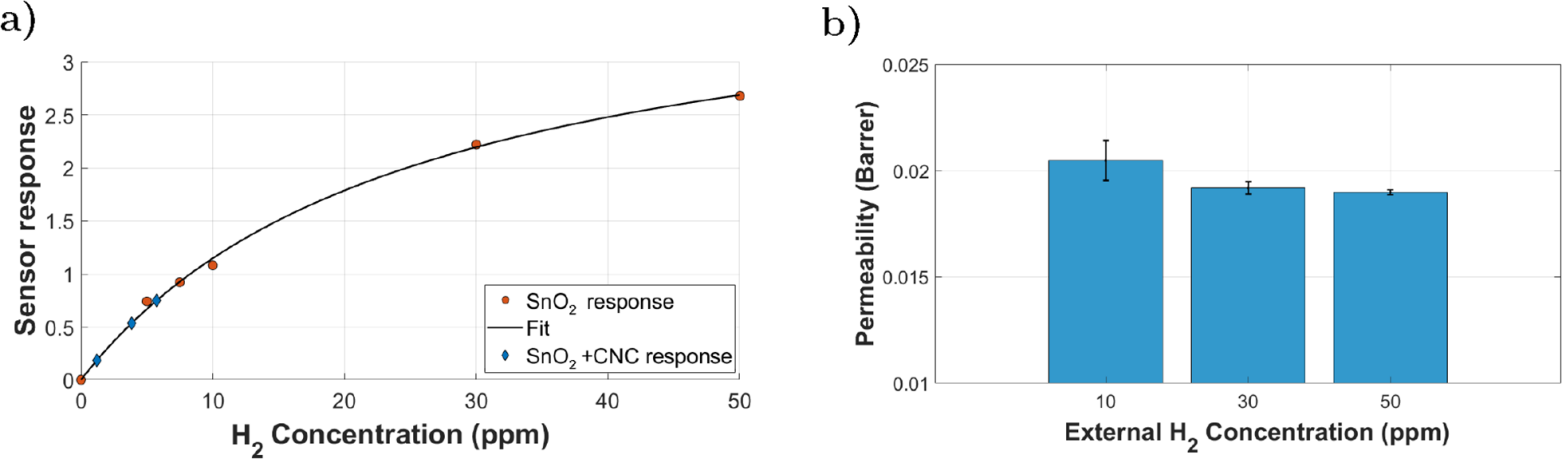
(a) Langmuir isotherm fitting for SnO_2_ response vs concentration alongside the data points used for the fitting in orange. The fitted equation was used to obtain concentration inside the membrane for SnO_2_ + CNC sensor shown in blue. (b) H_2_ permeability values for CNC membrane mounted on the sensor obtained at the 3 tested concentrations.

Knowing this internal concentration, the internal volume of the sensor *V*_int_, and the characteristic time (*T*_90_) over which the analyte builds up inside the housing, the flux *J* of gas entering through the membrane could be computed as

$$J_{90}=\frac{C\cdot V_{\mathrm{int}}\cdot 0.9}{T_{90}}$$ 6

From eq 6, knowing the internal area of the membrane housing and its thickness *d*, obtained by the density of CNCs, the deposition volume, and area, the permeability can be calculated as

$$\Phi_{\mathrm{sensor}}=\frac{J_{90}\cdot d}{\Delta P_{\mathrm{conc}}}$$ 7

with Δ*P*_conc_ being *H*_2_ partial pressure at a given concentration.

Although this method relies on the electrical signal of the SnO_2_ sensor rather than direct mass spectrometric detection, the resulting permeability values for H_2_ (Figure 11b) offer a reliable estimate of the transport properties of the membrane under working conditions. To validate these findings using an independent technique, direct permeability measurements were then performed using a QMS-based setup which is described in detail in the Materials and Methods. Once both sides of the system were flushed with the carrier and a stable baseline was established, the carrier on the retentate side was replaced with the analyte gas, introduced at a pressure 3 bar higher than the permeate side using a pressure controller. This pressure differential drove analyte transport through the membrane, and the permeated fraction was carried by a 1 sccm N_2_ flow to the QMS for detection. The pressure difference across the membrane drives the analyte through, and the fraction that permeates is carried by the carrier flux on the permeate side to the QMS for analysis. The permeation of CO_2_, O_2_, and N_2_ through the membrane was negligible and thus could not be reliably detected by QMS. In contrast, a detectable signal was obtained for H_2_ (Figure 12a) and He (Figure 12b), which depict the concentration over time in ppm inside the detector chamber for both gases.

**Figure 12:**
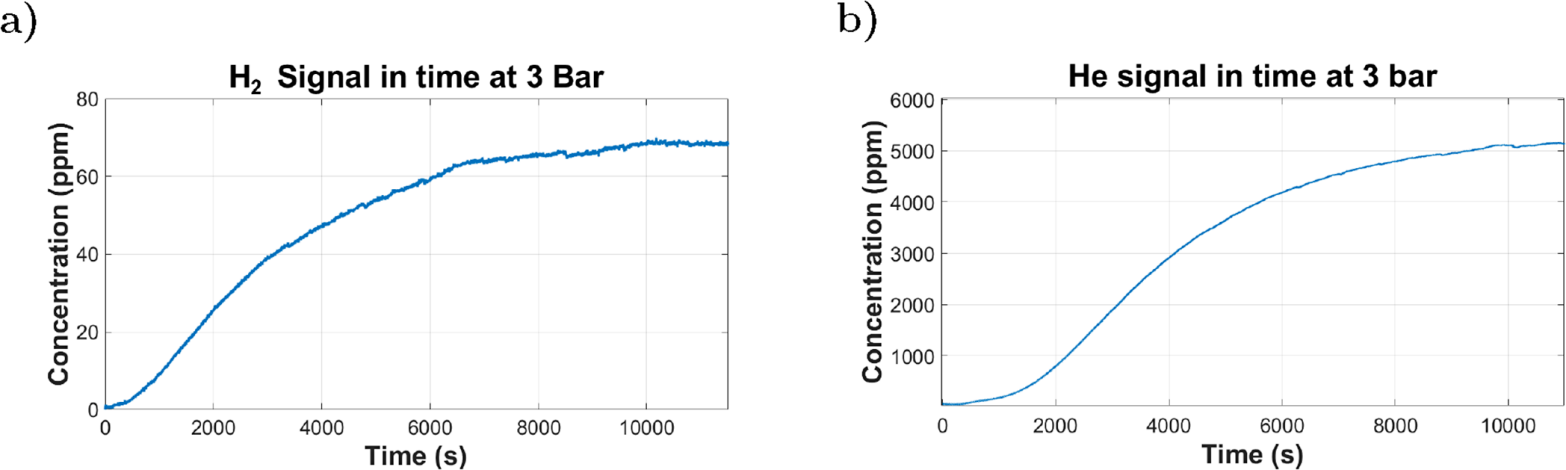
(a) H_2_ concentration in the detector over time. The analyte mixture contained only 4% H_2_; the maximum concentration detected in the permeate side was ∼70 ppm; thus, the concentration if 100% H_2_ was used would have been ∼1750 ppm. (b) He concentration in the detector over time. He, used as a pure analyte, reached a maximum concentration of ∼5160 ppm in the permeate side.

To quantify gas transport through the CNC membrane, the flux *J* can be determined as follows:

$$J=\frac{C\cdot Q}{A}$$ 8

with *C* the gas concentration in the carrier stream, *Q* the carrier flow rate and *A* the membrane area (18 cm^2^).

For He, which was fed at 100% concentration, the partial pressure difference Δ*P* across the membrane was simply the 3 bar. Consequently, knowing the measured membrane thickness *d* = 5.0 ± 0.6 μm, the permeability can be directly calculated using the equation reported below:

$$\Phi =\frac{J\cdot d}{\Delta P}$$ 9

In contrast, H_2_ was present at only 4% in its mixture, so the partial pressure driving force across the membrane is Δ*P*_H_2__ = 0.04 × 3 bar = 0.12 bar.

From this a value for H_2_ permeability of 0.018 ± 0.002 Barrer and a He permeability of 0.055 ± 0.007 Barrer could be obtained.

The permeability values obtained here differ from those reported in previous studies where much lower H_2_ permeation was observed.^30^ This discrepancy is likely due to different test conditions: the referenced work employed a constant volume–variable pressure system with ultrahigh vacuum on the permeate side, which can dehydrate the CNC membrane and significantly reduce its permeability. In contrast, the present QMS measurements were performed under near-ambient conditions, preserving membrane hydration and better reflecting real-world sensor operation. The agreement with permeability values derived from sensor response further supports the validity of the measurements under these conditions.

## Conclusions

This work demonstrates that organic membrane materials, specifically, cellulose nanocrystals (CNCs), can be effectively integrated into semiconducting metal oxide (SMOx) sensor platforms to substantially improve gas selectivity. The CNC membrane exhibited higher permeability toward H_2_, while significantly suppressing the permeation of interfering species such as acetone, EtOH, NH_3_, NO_2_ and CO.

To quantify the filtering performance of the membrane independently of the intrinsic selectivity of the SMOx sensor, a “barrier effect” metric was introduced, defined as the ratio between the response of the pristine sensor and that of the membrane-covered sensor under identical conditions. Under this framework, acetone transmission was suppressed by nearly a factor of 1700 at 50 ppm, and EtOH by a factor of approximately 190, confirming strong size and polarity dependent selectivity. In contrast, H_2_ permeation remained largely unaffected, preserving its sensor response and thereby enhancing effective H_2_ selectivity.

Direct permeation measurements using a QMS confirmed the permeability of the membrane to H_2_, yielding a value of approximately 0.018 ± 0.002 Barrer. The present results, obtained under near-ambient conditions were consistent with the independent sensor-based estimation.

Overall, the findings highlight a practical and low-cost strategy to improve SMOx sensor selectivity through the application of a solution-processable, bioderived membrane. By retaining high intrinsic sensitivity while selectively filtering larger or more polar analytes, this membrane-on-package approach offers a viable pathway toward hybrid sensor–membrane systems capable of addressing the selectivity limitations of low-cost gas sensors. Despite these promising results, some limitations remain. The CNC membrane introduces a slight reduction in the H_2_ signal, as well as longer response and recovery times (*T*_90_ and *T*_10_) across most analytes, due to the added transport barrier. These trade-offs must be considered when designing applications requiring rapid detection or minimal signal attenuation.

Future work will focus on minimizing these drawbacks through membrane optimization. Reducing membrane thickness, refining deposition protocols, and tailoring the placement within the sensor assembly may help strike a better balance between selectivity, sensitivity, and response time. In parallel, efforts toward integrating this concept into miniaturized sensing platforms will be crucial for enabling real-world deployment of membrane-enhanced SMOx sensors in compact, low-power devices.

## Supplementary Material



## Notes

Additional experimental details and methods, including sensor and gas-permeation setup descriptions and photographs, CNC membrane characterization (ζ-potential, UV–vis transmittance, tensile tests), SnO_2_ sensing-layer SEM/EDX, barrier-effect analyses versus concentration and device aging, LOD calculations, and response dynamics (*T*_10_/*T*_90_) (PDF)
