# Supplementary material for: Improving Hydrogen Selectivity in Semiconductor Metal Oxide Gas Sensors with Cellulose Nanocrystal Membranes
Source: ACS Sens. 2025 Oct 16;10(11):8411–23. doi: 10.1021/acssensors.5c01801 (PMC12670992; doi:10.1021/acssensors.5c01801)
Supplement: Supplementary file 1 [file se5c01801_si_001.pdf]

Improving Hydrogen Selectivity in Semiconductor Metal Oxide Gas Sensors with Cellulose Nanocrystal Membranes

Guglielmo Trentini,[*,†,‡] Antonio Orlando,[†,‡] Soufiane Krik,[†] Pietro Tosato,[‡] Matteo Valt,[‡] Luisa Petti,[†] Marina Scarpa,[¶] and Andrea Gaiardo[‡]

†*Faculty of Engineering, Free University of Bolzano-Bozen, Via Bruno Buozzi, 1, 39100 Bolzano, Italy*

‡*Materials and Topologies for Sensors and Devices, Sensors and Devices Center, Bruno Kessler Foundation, Via Sommarive 18, 38123 Trento, Italy*

¶*Department of Physics, University of Trento, Via Sommarive, 14, 38123 Povo, Trento, Italy*

E-mail: gtrentini@fbk.eu

Supporting Info

Setup description

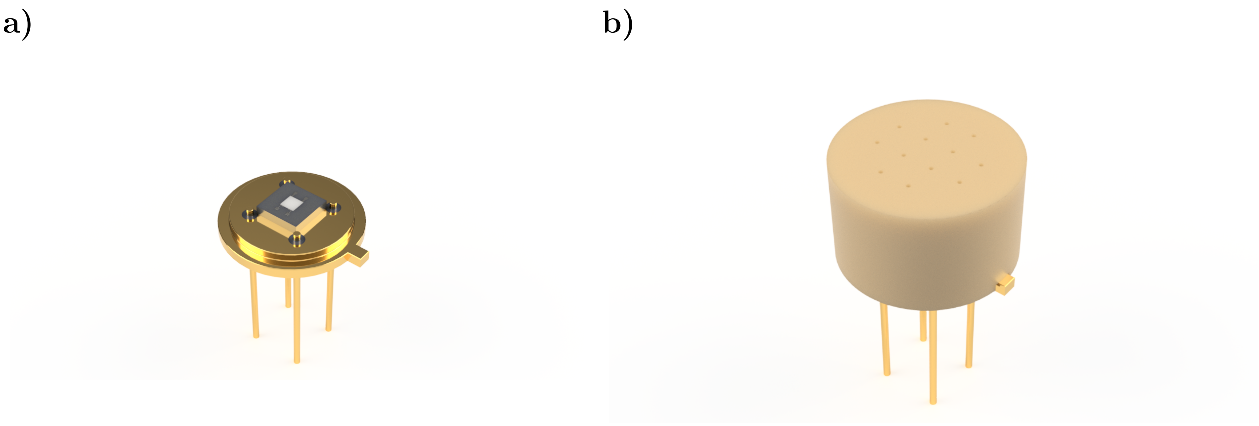

Figure 1: Sensor assembly before (a) and after (b) CNC membrane integration. (a) Shows the bare sensor mounted on the TO-39, while (b) displays the final device with the CNC membrane in the custom PEEK cap

Figure 1 shows the sensor platform used in this study, both before and after integration of the CNC membrane. The membrane was embedded in a custom-machined PEEK cap, which was then mounted onto a standard TO-39 package to seal the sensing layer. This packaging approach allows straightforward handling and facilitates integration into various test environments.

The sensors were placed side by side inside a measurement chamber and connected to a custom PCB via sealed through-holes for power supply and resistance readout. A stable baseline was established using synthetic air (80% N_2 and 20% O_2) flowing at 200 sccm, controlled via mass flow controllers (MFCs). Certified analyte gas mixtures were diluted with synthetic air to reach desired concentrations. The system was fully automated via an in-house LabVIEW program, coordinating MFCs, gas injection, and data recording.

To complement sensor-based estimates and independently validate the transport properties of the CNC membrane, gas permeability was also evaluated using a dedicated analytical setup. While the previous measurements relied on sensor signal modulation, this configuration allowed direct quantification of permeated gases under controlled conditions.

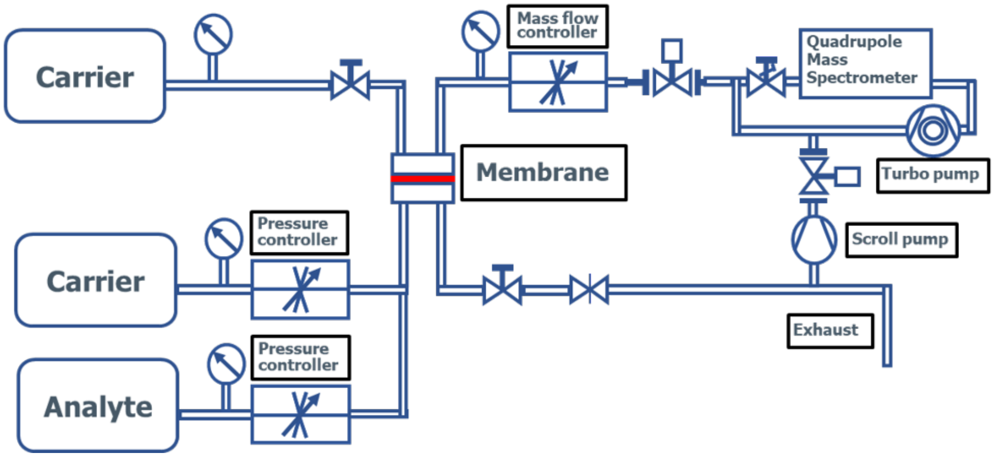

Figure 2: Constant pressure–variable volume setup used for permeability experiments. The CNC membrane is positioned in the permeation cell between two gas flows. On the permeate side, a carrier gas (N_2) flux of 1 sccm transports permeated gases to the quadrupole mass spectrometer (QMS), which monitors their concentration in time.

The setup, shown in Figure 2, is based on a constant pressure–variable volume approach. The membrane is mounted in a dedicated permeation cell separating two gas lines. A high-pressure analyte stream is introduced on the retentate side, while a low-flow (1 sccm) N_2 carrier on the permeate side collects any transmitted species and carries them to a quadrupole mass spectrometer (QMS) for detection. The reduced flow improves detection sensitivity by increasing analyte concentration at the detector. The QMS continuously monitors the concentration of permeated species, enabling extraction of steady-state permeability values. Due to the limited flux through the relatively thick membrane and the inherent dead time of the system, transient measurements were not used to calculate diffusivity, and only steady-state values were considered reliable.

CNC membrane characterization

To evaluate the suitability of the CNC membrane for integration with the sensor the suspension was analyzed in terms of colloidal stability, surface functionalization, and particle morphology. Zeta potential measurements revealed a strongly negative surface charge of –64.7 ± 3.5 mV, confirming excellent dispersion stability as a result of the successful introduction of carboxylic groups through TEMPO-mediated oxidation.

a) b)

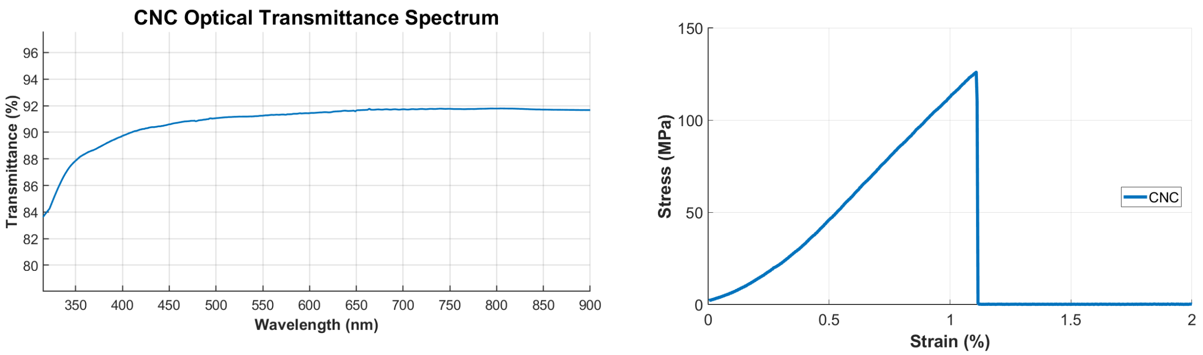

Figure 3: a) Optical transmittance spectrum of the CNC membrane. The film exhibits over 90% transmittance above 400 nm, indicating high transparency across the visible range. b) Representative stress–strain curve of the CNC membrane, showing a stress at break of 125 MPa at 1.1% strain.

To assess the optical properties of the CNC membrane, UV–vis transmittance measurements were performed in the 320–900 nm range. As shown in Figure 3a, the membrane exhibited high transparency across the visible spectrum, with transmittance exceeding 90% above 400 nm. The high transparency of the CNC membrane suggests potential compatibility even with gas sensors that operate via optical or photoactivated mechanisms, rather than thermal activation.

Before integrating them with the sensor, the membranes were tested mechanically to ensure they could handle typical operating conditions. Mechanical characterization of the CNC films showed a stress at break of 141 ± 17 MPa and an elongation at break of 1.3 ± 0.5%, indicating sufficient robustness to withstand handling and the pressure differentials encountered in typical sensor housings (Figure 3b).

AFM images performed on dilute CNC deposition revealed a well-defined needle-like morphology. Image analysis of 50 individual crystals yielded an average length of 206±50 nm and a height of 5.6±1.4 nm, confirming successful fiber degradation and nanoscale uniformity of the CNC.

Sensing layer characterization In addition to the electrical characterization of the bare SnO_2 sensor used as a reference in this work, further analyses were carried out to evaluate the morphology and purity of the sensing layer. SEM was used to inspect the surface

of the printed film and confirmed that the deposition was uniform and exhibited a porous microstructure suitable for gas diffusion and adsorption (Figure 4a). EDX was performed to assess the elemental composition of the material and verify the successful removal of organic ligands after thermal treatment. The EDX spectrum (Figure 4b) confirmed the dominant presence of tin and oxygen and the atomic ratio was consistent with the expected SnO_2 stoichiometry, confirming the formation of a clean, fully oxidized sensing layer. Overall, the SEM and EDX results indicate a standard SnO_2 nanoparticle deposition typically used for gas sensing, with no unexpected morphological or compositional anomalies.

a)

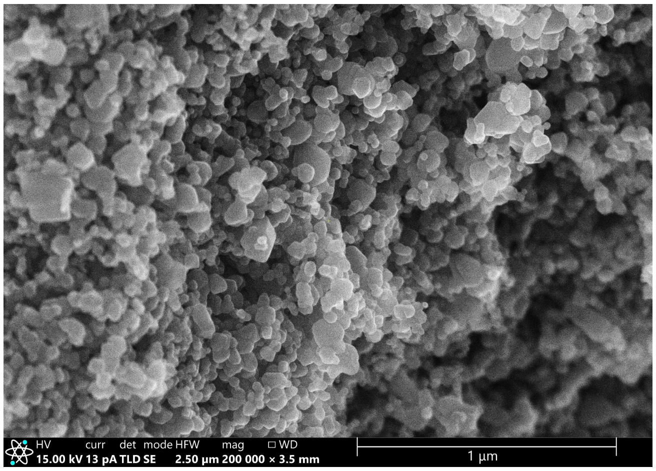

b)

Element Number	Element Symbol	Element Name	Atomic Conc. [%]	Weight Conc. [%]
8	O	Oxygen	73.88	27.60
50	Sn	Tin	26.12	72.40

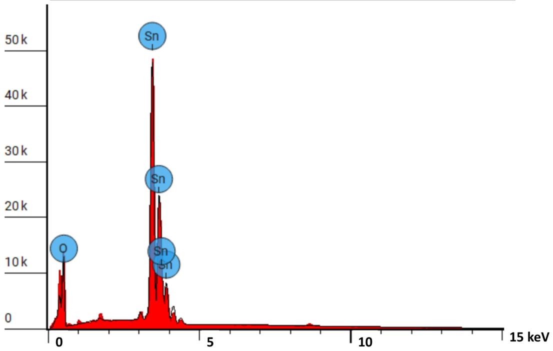

Figure 4: a) SEM image of the commercial SnO_2 sensing layer, showing uniform deposition with interconnected nanoparticles and high exposed surface area. (b) EDX spectrum and elemental composition confirming the exclusive presence of Sn and O, with no detectable contaminants.

Barrier Effect vs. Concentration

The barrier effect trends help to elucidate how the CNC membrane suppresses the responses of different gases based on their molecular size, polarity, and specific chemical interactions. *Acetone* exhibits the strongest barrier effect among all tested analytes. Its large kinetic diameter and high polarity contribute to both steric hindrance and strong interactions with the functional groups of the membrane. The fact that the barrier effect increases with concentration suggests that diffusion becomes increasingly restricted at higher loading,

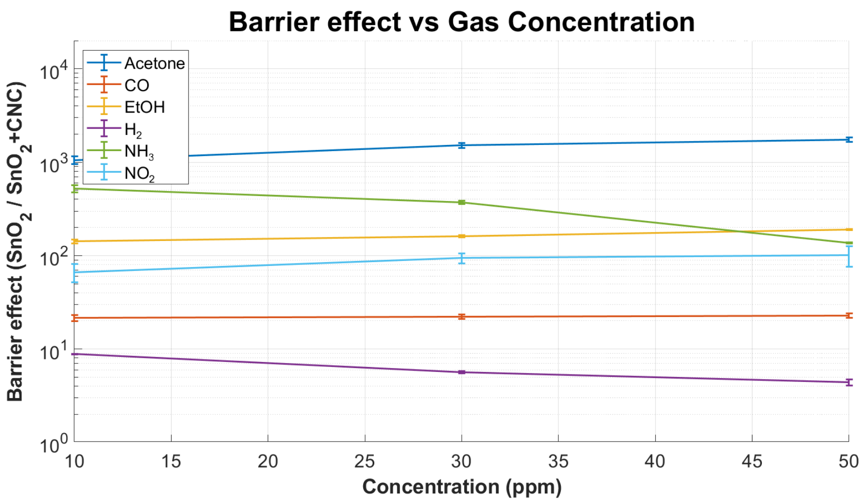

Figure 5: Barrier effect ($\text{Response}_{SnO_2}/\text{Response}_{SnO_2+CNC}$) as a function of concentration. Higher values indicate greater suppression by the CNC membrane.

possibly due to limited membrane permeability for such large, polar molecules.

$EtOH$ also experiences a significant barrier effect, though to a lesser extent than acetone. Its slightly smaller size allows for more facile diffusion, while its moderate polarity ensures some degree of interaction with the membrane. The near-linear increase in barrier effect with concentration implies that ethanol diffusion is more proportionally related to the driving concentration gradient, with less pronounced bottlenecks compared to acetone.

CO shows a minimal and stable barrier effect across all tested concentrations. This is attributed to its small molecular size and very low polarity, resulting in negligible interactions with the CNC membrane. Its permeation is predominantly governed by passive diffusion rather than adsorption-related effects.

NH_3 presents a more complex behavior: a high barrier effect at low concentrations, which gradually decreases as concentration increases. This suggests that at lower concentrations, NH_3 strongly interacts with the membrane, likely through hydrogen bonding with carboxylic groups or simple substitution reactions effectively suppressing its passage. However, as concentration rises, these adsorption sites become saturated, and diffusion starts to dominate, resulting in increased permeation. Despite its small molecular size, NO_2 exhibits a notable and increasing barrier effect with concentration. This trend is attributed to its relatively

high polarity, which promotes strong interactions with the carboxylated CNC membrane. As concentration increases, these polar interactions likely become more pronounced, enhancing the selective hindrance of the membrane to NO_2 permeation. This behavior demonstrates that for highly interactive gases such as NO_2 and NH_3, suppression is not solely governed by size but also by the strength of physicochemical interactions with the membrane. Hydrogen, as expected, exhibits very low barrier effect values. Its extremely small kinetic diameter and nonpolar nature allow it to readily permeate the membrane. A slight decrease in barrier effect with increasing concentration may indicate minimal adsorption effects at low concentrations which become negligible at higher loading. This behavior aligns with the design objective of the membrane, preserving hydrogen sensitivity while reducing interference from larger or more interactive analytes.

Barrier effect over time

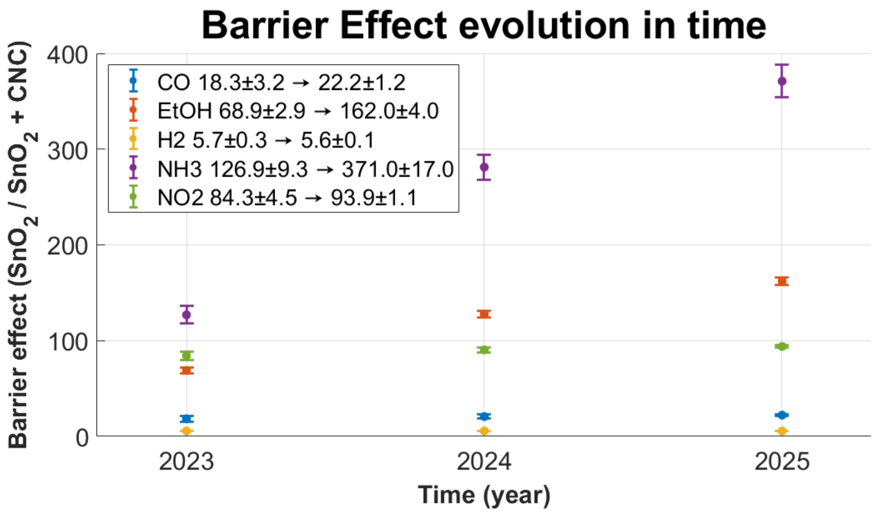

Figure 6: Barrier effect ($\text{Response}_{\text{SnO}_2}/\text{Response}_{\text{SnO}_2+\text{CNC}}$) at three time points after device fabrication. Markers show mean $\pm$ SD;. The barrier effect for interferants is maintained or increases over time, while H_2 is nearly constant.

To assess stability, the barrier effect was measured at three snapshots: (1) shortly after fabrication (2023), (2) mid-2024, and (3) 2025. Across interferants (EtOH, NH_3, NO_2, CO) the barrier effect is stable or increases with time, whereas the value for H_2 remains essentially unchanged. This trend is consistent with the well-known phenomena of membrane

conditioning/aging, where overall permeability gradually decreases with time due to physical relaxations; because the H_2 barrier effect is essentially constant while interferants show equal or greater suppression, the net selectivity to H_2 increases over time.

Limit of Detection (LOD)

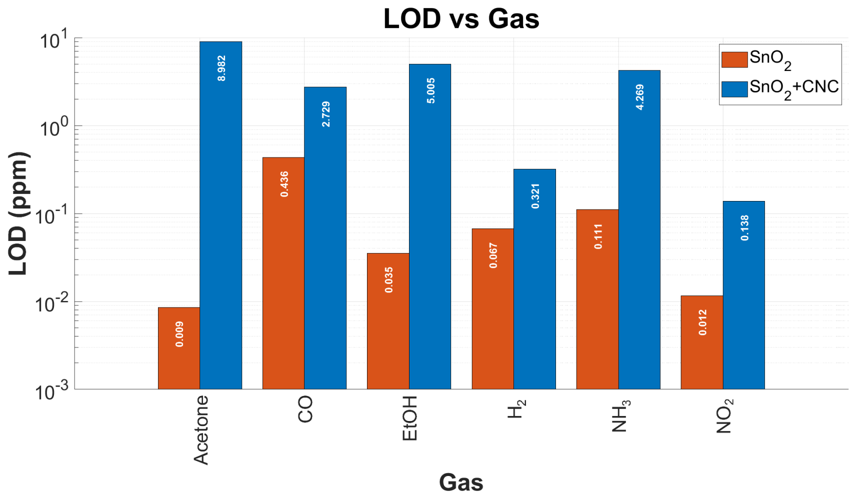

Figure 7: LOD increase for each gas due to the CNC membrane. Acetone shows the greater change due to the high barrier effect towards it while H_2 has the lowest increase

Table 1: LOD values for SnO_2 and SnO_2 + CNC sensors and the resulting increase.

Gas	LOD (SnO_2 + CNC) (ppm)	LOD (SnO_2) (ppm)	LOD Increase
Acetone	8.98	0.0085	1055.07
CO	2.73	0.44	6.26
EtOH	5.00	0.0353	141.94
H_2	0.32	0.067	4.80
NH_3	4.27	0.11	38.59
NO_2	0.14	0.012	11.86

LOD increases are consistent with barrier effect trends: larger, more polar molecules experience stronger suppression and thus higher LOD values, while smaller or less interactive gases like CO, NO_2, and H_2 are less affected. This confirms that the CNC membrane enhances selectivity primarily via steric and polarity-based mechanisms without drastically compromising H_2 detection.

T10 and T90 Dynamics

a) b)

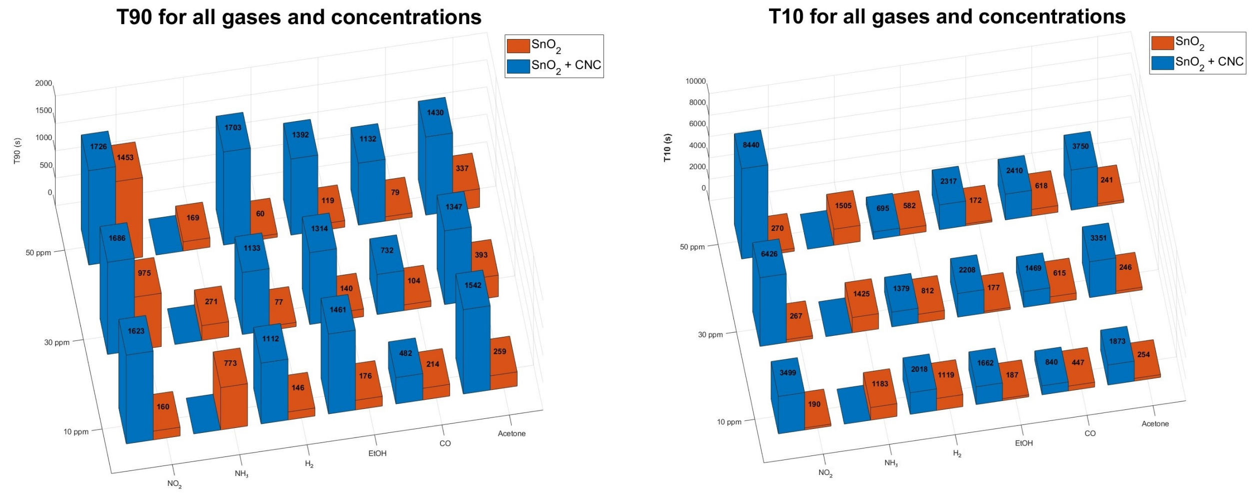

Figure 8: T90 a) and T10 b) times (log scale) for all tested gases at various concentrations. For NO_2, actual concentrations were 1, 3, and 5 ppm. NH_3 times could not be measured reliably due to the transient oxidizing behavior observed.

The addition of the CNC membrane increases both T90 and T10 values across all gases, consistent with the introduction of a diffusion barrier. Gases with stronger interactions with the membrane, such as $EtOH$ and CO, exhibit more pronounced delays. NH_3 dynamics could not be accurately extracted due to low response intensity and transient oxidation artifacts. In contrast, H_2, while also affected, maintains comparatively faster dynamics. These findings demonstrate a trade-off between enhanced selectivity and slower sensor response, with the extent of delay depending on gas-specific properties like size and polarity.
